# Supplementary material for: The association between the extracellular water-to-total body water ratio and albuminuria in Chinese type 2 diabetes mellitus patients
Source: PeerJ. 2025 Jul 31;13:e19780. doi: 10.7717/peerj.19780 (PMC12318500; doi:10.7717/peerj.19780)
Supplement: Supplemental Information 1 [file peerj-13-19780-s001.docx]

| **Supplementary Table** Comparison of the ECW/TBW ratio across various body regions among three patient groups. | | | | | |
| --- | --- | --- | --- | --- | --- |
| Body parts | Total | Normo-albuminuria | Microalbuminuria | Macroalbuminuria | *P* |
| n | 1034 | 640 | 244 | 150 |  |
| Right arms, n (%) |  |  |  |  | 0.015 |
| Low | 502 (48.55) | 327 (51.09) | 118 (48.36) | 57 (38.00) |  |
| High | 532 (51.45) | 313 (48.91) | 126 (51.64) | 93 (62.00) |  |
| Left arms, n (%) |  |  |  |  | 0.002 |
| Low | 491 (47.49) | 327 (51.09) | 111 (45.49) | 53 (35.33) |  |
| High | 543 (52.51) | 313 (48.91) | 133 (54.51) | 97 (64.67) |  |
| Trunk, n (%) |  |  |  |  | <0.001 |
| Low | 488 (47.20) | 321 (50.16) | 121 (49.59) | 46 (30.67) |  |
| High | 546 (52.80) | 319 (49.84) | 123 (50.41) | 104 (69.33) |  |
| Right legs, n (%) |  |  |  |  | <0.001 |
| Low | 498 (48.16) | 332 (51.88) | 119 (48.77) | 47 (31.33) |  |
| High | 536 (51.84) | 308 (48.12) | 125 (51.23) | 103 (68.67) |  |
| Left legs, n (%) |  |  |  |  | <0.001 |
| Low | 487 (47.10) | 322 (50.31) | 120 (49.18) | 45 (30.00) |  |
| High | 547 (52.90) | 318 (49.69) | 124 (50.82) | 105 (70.00) |  |
